# Comparison of the efficacy and safety of low-dose calcineurin inhibitors plus sirolimus plus mycophenolic acid with the standard-dose calcineurin inhibitors plus mycophenolic acid regimen in patients who received kidney transplants

**DOI:** 10.3389/fphar.2025.1631055

**Published:** 2025-09-08

**Authors:** Li Li, Jiao Wan, Yizhi Li, Jiali Fang, Guanghui Li, Junjie Ma, Zheng Chen

**Affiliations:** ^1^ Department of Organ Transplantation, The Second Affiliated Hospital of Guangzhou Medical University, Guangzhou, China; ^2^ Department of Urology, Maoming People’s Hospital, Maoming, China

**Keywords:** calcineurin inhibitors, sirolimus, kidney transplant, renoprotective, safety

## Abstract

**Background:**

Sirolimus (SRL) has shown its anti-rejection and renoprotective efficacy in patients with kidney transplantation. However, more evidence is still needed. The current study aimed to evaluate the efficacy and safety of an SRL-containing regimen in patients who received kidney transplants.

**Methods:**

Fifty patients with end-stage renal disease who received kidney transplants were enrolled and divided into the calcineurin inhibitors (CNI) + mycophenolic acid (MPA)+ glucocorticoid (*N =* 22) and CNI + MPA + SRL + glucocorticoid groups (*N =* 28) according to the actual regimen that they received. The minimal plasma concentration of tacrolimus and cyclosporin was maintained at 6–10 ng/mL and 150–250 ng/mL in the CNI + MPA + glucocorticoid group and 4–6 ng/mL and 75–125 ng/mL in the CNI + MPA + SRL + glucocorticoid group. The minimal plasma concentration of SRL was maintained at 5–8 ng/mL.

**Results:**

The Cr at month (M)6, M12, and uric acid at M3 were lower, while the eGFR at M12 was higher in the CNI + MPA + SRL + glucocorticoid group compared with the CNI + MPA + glucocorticoid group (all *P* < 0.05). The acute rejection rate showed a lower trend in the CNI + MPA + SRL + glucocorticoid group compared with the CNI + MPA + glucocorticoid group without statistical significance. The urine BK virus at M3, M6, M9, and M12 was lower in the CNI + MPA + SRL + glucocorticoid group compared with the CNI + MPA + glucocorticoid group (all *P* < 0.05). Incidence of most adverse events was similar between groups, except that BK virus was lower in the CNI + MPA + SRL + glucocorticoid group compared with the CNI + MPA + glucocorticoid group (0.0% vs. 36.4%, *P* < 0.01).

**Conclusion:**

Low-dose CNI combined with SRL regimen as the initial anti-rejection regimen indicates a comparable anti-rejection and better renoprotective efficacy with a satisfying safety profile compared with the standard regimen.

## 1 Introduction

Kidney transplantation is the most promising therapeutic approach for patients with end-stage renal disease (ESRD), which prolongs survival and improves the quality of life compared to long-term dialysis (Abramyan and Hanlon, 2025; Dash et al., 2023; Kostro et al., 2016; Molnar et al., 2016). During the post-transplantation period, anti-rejection therapy plays an important role in determining the graft and patients’ survival (Khan et al., 2025; Shokeir et al., 2021). Calcineurin inhibitors (CNIs), represented by tacrolimus and cyclosporine, are commonly used in preventing acute rejection post transplantation (Kajdas et al., 2025; Mok et al., 2025). Despite their efficacy, it should not neglected that the CNIs might induce the serious side effects, such as the nephrotoxicity, which would finally cause the graft loss and other adverse events including the hypertension, hyperlipidemia, and post-transplant malignancies (Hansen et al., 2025; Oliveras et al., 2024). Therefore, it is urgent to explore the alternative immunosuppressive drugs with fewer adverse events in these patients.

Rapamycin (sirolimus, SRL) is an mTOR inhibitor, which shows the reduced nephrotoxicity and anti-proliferative properties; therefore, it is considered to be a promising alternative to CNIs (Chen et al., 2013; Préterre et al., 2021). In recent studies, SRL combined with CNIs might help to reduce the dosage of CNIs, which subsequently reduces the incidence of post-transplant adverse events and improves graft survival. For instance, it is shown that converting to an SRL-containing regimen might elevate the renal function in patients after kidney transplantation (Pankewycz et al., 2011). Apart from that, the anti-BK virus of sirolimus should also be noticed (Liacini et al., 2010). In recent studies, it indicates that the SRL might reduce the BK virus to 4.3%, which is lower than the 17.9% of tacrolimus in kidney transplant recipients (Tohme et al., 2015). However, the evidence about applying the SRL-containing regimen in post-kidney-transplant patients is still inadequate, which largely affects its clinical application.

Hence, the current study aimed to evaluate the efficacy and safety of an SRL-containing regimen in patients who received kidney transplants.

## 2 Materials and methods

### 2.1 Patients

Fifty patients with end-stage renal disease who received kidney transplants from 26 April 2022 to 11 October 2023 were enrolled in the prospective study. The inclusion criteria contained: (1) end-stage renal disease patients; (2) aged 18–70 years; (3) firstly received kidney transplants from deceased donors or living donors; (4) preoperative panel reactive antibody ≤10%; (5) estimated glomerular filtration rate (eGFR) ≥40 mL/min/1.73 m^2^. The exclusion criteria contained: (1) had active infections; (2) had infiltrates, cavitation, or consolidation by chest X-ray; (3) had focal segmental glomerulosclerosis or membranoproliferative glomerulonephritis; (4) underwent multi-organ transplants; (5) fasting triglyceride ≥4.6 mmol/L and/or cholesterol ≥7.8 mmol/L; (6) 24-h urinary protein excretion >500 mg daily; (7) had active hepatitis B or C infections; (8) had human immunodeficiency virus (HIV) seropositivity; (9) used other investigational drugs before enrollment; (10) had malignancy histories within 3 years (excluding well-treated basal cell carcinoma and squamous cell carcinoma); (11) donation from monozygotic twins; (12) intended to use substances with strong interactions of SRL; (13) pregnant, planning pregnancy, or breastfeeding females; (14) had any conditions deemed clinically inappropriate for participation by the investigators. The study was permitted by the Ethics Committee (2021-hs-77). All patients offered written informed consent.

### 2.2 Treatments

Based on the different treatment regimens after kidney transplants, the patients were divided into CNI + mycophenolic acid (MPA) + glucocorticoid and CNI + MPA + SRL + glucocorticoid groups.

For the CNI + MPA + glucocorticoid group, patients received CNI (tacrolimus (TAC) or cyclosporin (CsA)), MPA (mycophenolate mofetil (MMF) or enteric-coated mycophenolate sodium (EC-MPS)), and glucocorticoid (prednisone and methylprednisolone) until 1 year after kidney transplants. The minimal plasma concentration of TAC was recommended as 6–10 ng/mL, CsA as 150–250 ng/mL, the dose of MMF as 1–2 g/d, EC-MPS as 1,080–1,440 mg daily, and prednisone as 5–10 mg daily. Then, patients continually received CNI, MPA, and glucocorticoid until 2 years after kidney transplants. The minimal plasma concentration of TAC was recommended as 5–7 ng/mL, and CsA as 100–200 ng/mL. The doses of MMF, EC-MPS, and prednisone were maintained.

For the CNI + MPA + SRL + glucocorticoid group, patients received CNI, MPA, and glucocorticoid with the same dosage of CNI + MPA + glucocorticoid group until 2 weeks (W2) after kidney transplants. Then, patients received low-dose CNI, SRL, MPA, and glucocorticoid until 1 year after kidney transplants. The TAC dose was reduced by 1/3, resulting in the minimal plasma concentration dropping to 4–6 ng/mL; the CsA dose was reduced by 1/2, leading to the minimal plasma concentration falling to 75–125 ng/mL. The dose of SRL was 1–2 mg/Qd, and the minimal plasma concentration was 5–8 ng/mL (Association, 2018). The dose of MMF was 0.5–1 g/d, EC-MPS of 1,080–1,440 mg daily, and prednisone of 5–10 mg daily. Subsequently, patients received low-dose CNI, SRL, MPA, and glucocorticoid until 2 years after kidney transplants. The minimal plasma concentration of TAC was reduced to 3–5 ng/mL; the CsA was reduced to 50–100 ng/mL. The doses of SRL, MMF, EC-MPS, and prednisone were maintained.

### 2.3 Assessments

To closely monitor the therapeutic effect and adverse events, the biochemical markers were assessed. The creatinine (Cr), eGFR, uric acid, urine erythrocyte, urine leukocytes, urine BK virus, blood BK virus, alanine aminotransferase (ALT), aspartate aminotransferase (AST), cholesterol, triglyceride, white blood cell count (WBC), hemoglobin (HGB), and platelet (PLT) were collected at W2, 3 months (M3), 6 months (M6), 9 months (M9), and 12 months (M12) after kidney transplants. Besides, the incidence of acute rejection and adverse events within 2 years after kidney transplants were also collected.

### 2.4 Statistics

SPSS version 26.0 was utilized for data analyses. The comparisons between groups were completed using Student’s t-test, Wilcoxon rank sum test, Chi-square test, or Fisher’s exact test. *P* < 0.05 was considered significant.

## 3 Results

### 3.1 Patients characteristics

The age was 41.2 ± 13.1 years in the CNI + MPA + SRL + glucocorticoid group, consisting of 18 (64.3%) males and 10 (35.7%) females. In the CNI + MPA + glucocorticoid group, the age was 39.3 ± 9.1 years, with 14 (63.6%) males and 8 (36.4%) females. In terms of the type of end-stage renal disease, 11 (64.7%) patients were diagnosed with chronic glomerulonephritis, 1 (5.9%) patient diagnosed as IgA nephropathy, 2 (11.8%) patients diagnosed as obstructive nephropathy, 2 (11.8%) patients diagnosed as hypertensive nephropathy, and one patient diagnosed as other renal disease in the CNI + MPA + SRL + glucocorticoid group. However, in the CNI + MPA + glucocorticoid group, there were respectively 13 (61.9%), 4 (19.0%), 1 (4.8%), 2 (9.5%), and 1 (4.8%) patients who were diagnosed as chronic glomerulonephritis, IgA nephropathy, obstructive nephropathy, polycystic kidney, and other renal disease (*P* = 0.028). Regarding the type of dialysis, there were 8 (47.1%), 6 (35.3%), and 3 (17.6%) patients receiving the hemodialysis, peritoneal dialysis, and hemodialysis and peritoneal dialysis, respectively in the CNI + MPA + SRL + glucocorticoid group; these proporations were 16 (76.2%), 3 (14.3%), and 2 (9.5%) in the CNI + MPA + glucocorticoid group (*P* = 0.009). By comparison, other characteristics, including the age, sex, body mass index, smoke history, drink history, concomitant diseases, and duration of dialysis were not different between these two groups (All *P* > 0.05, Table 1).

**TABLE 1:** Clinical characteristics of patients.

Items	CNI + MPA + SRL + glucocorticoid	CNI + MPA + glucocorticoid	P-value
Age (years)			
Patients (Missing)	28 (0)	22 (0)	
Mean (SD)	41.2 (13.1)	39.3 (9.1)	0.243
Median	39.0	35.0	
Min, max	22.0, 73.0	28.0, 55.0	
Sex			
Patients (Missing)	28 (0)	22 (0)	
Male, n (%)	18 (64.3)	14 (63.6)	0.962
Female, n (%)	10 (35.7)	8 (36.4)	
Body mass index (kg/m^2^)			
Patients (Missing)	17 (11)	21 (1)	
Mean (SD)	22.3 (3.7)	21.5 (4.1)	0.517
Median	22.3	20.7	
Min, max	17.3, 31.9	14.6, 32.8	
Smoke history			
Patients (Missing)	17 (11)	21 (1)	
No, n (%)	16 (94.1)	16 (76.2)	0.132
Yes, n (%)	1 (5.9)	5 (23.8)	
Drink history			
Patients (Missing)	17 (11)	21 (1)	
No, n (%)	15 (88.2)	19 (90.5)	0.823
Yes, n (%)	2 (11.8)	2 (9.5)	
Concomitant diseases			
Patients (Missing)	17 (11)	21 (1)	
Anemia, n (%)	16 (94.1)	21 (100.0)	0.447
Hypertension, n (%)	17 (100.0)	21 (100.0)	-
Diabetes, n (%)	1 (5.9)	0 (0.0)	0.447
Coronary heart diseases, n (%)	0 (0.0)	0 (0.0)	-
Hyperlipidemia, n (%)	0 (0.0)	2 (9.5)	0.492
Hyperuricemia, n (%)	1 (5.9)	7 (33.3)	0.053
Hepatitis B, n (%)	2 (11.8)	0 (0.0)	0.193
Others, n (%)	10 (58.8)	13 (61.9)	0.847
Type of end-stage renal disease			
Patients (Missing)	17 (11)	21 (1)	0.028
Chronic glomerulonephritis, n (%)	11 (64.7)	13 (61.9)	
IgA nephropathy, n (%)	1 (5.9)	4 (19.0)	
Obstructive nephropathy, n (%)	2 (11.8)	1 (4.8)	
Polycystic kidney, n (%)	0 (0.0)	2 (9.5)	
Hypertensive nephropathy, n (%)	2 (11.8)	0 (0.0)	
Others, n (%)	1 (5.9)	1 (4.8)	
Type of dialysis			
Patients (Missing)	17 (11)	21 (1)	0.009
Hemodialysis, n (%)	8 (47.1)	16 (76.2)	
Peritoneal dialysis, n (%)	6 (35.3)	3 (14.3)	
Hemodialysis and peritoneal dialysis, n (%)	3 (17.6)	2 (9.5)	
Duration of dialysis (months)			
Patients (Missing)	17 (11)	21 (1)	
Mean (SD)	20.0 (16.0)	23.8 (24.4)	0.589
Median	12.0	12.0	
Min, max	6.0, 60.0	0.8, 84.0	
Regimen of glucocorticoids			
Methylprednisolone tablet, n (%)	7 (25.0)	2 (9.1)	0.266
Prednisone, n (%)	21 (75.0)	20 (90.9)	

There was no statistical significance between two groups.

CNI, calcineurin Inhibitor; MPA, mycophenolic acid; SRL, sirolimus; SD, standard deviation.

### 3.2 Comparison of the renal function and uric acid between groups

**FIGURE 1:**
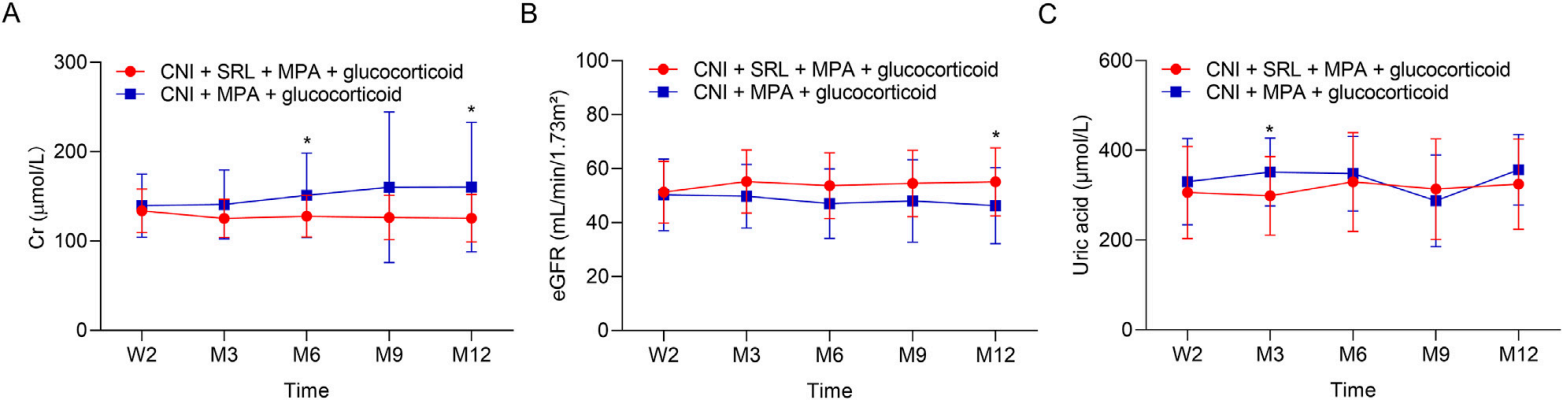
The renal function and uric acid level between groups. Comparison of the Cr **(A)**, eGFR **(B)**, and uric acid **(C)** between the CNI + MPA + SRL + glucocorticoid and the CNI + MPA + glucocorticoid groups.

The Cr was lower in the CNI + MPA + SRL + glucocorticoid group compared with the CNI + MPA + glucocorticoid group at M6 and M12 (both *P* < 0.05), while it remained unchanged at W2, M3, and M9 between groups (all *P* > 0.05) (Figure 1A). Chronologically, the Cr was gradually increased in the CNI + MPA + glucocorticoid group during the 12 months follow-up, while in the CNI + MPA + SRL + glucocorticoid group, the Cr decreased from W2 to M3, and it remained the steady state from M3 to M12. The eGFR was higher in the CNI + MPA + SRL + glucocorticoid group compared with the CNI + MPA + glucocorticoid group at M12 (*P* < 0.05), while it was similar between groups at W2, M3, M6, and M9 (all *P* > 0.05) (Figure 1B). Chronologically, the eGFR was continuously decreased in the CNI + MPA + glucocorticoid group during the 12 months follow-up, while in the CNI + MPA + SRL + glucocorticoid group, the eGFR increased from W2 to M3, and it remained the steady state from M3 to M12. In terms of the uric acid, it was lower in the CNI + MPA + SRL + glucocorticoid group compared with the CNI + MPA + glucocorticoid group at M3 (*P* < 0.05), while it was not different at other follow-up timepoints (all *P* > 0.05) (Figure 1C).

### 3.3 Comparison of the acute rejection rate between groups

**FIGURE 2:**
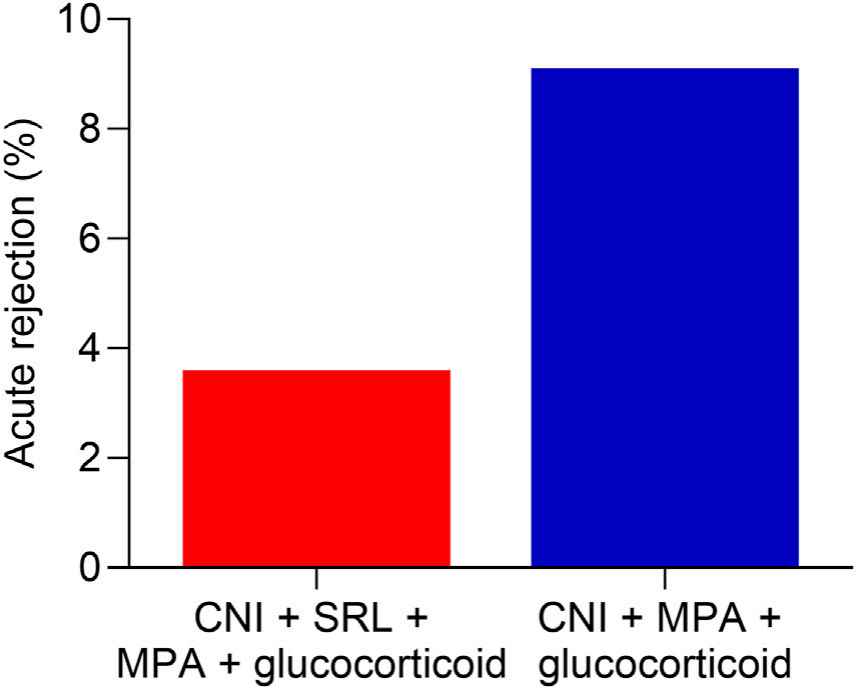
The acute rejection rate between groups. Comparison of acute rejection rate between the CNI + MPA + SRL + glucocorticoid and the CNI + MPA + glucocorticoid groups.

The acute rejection rate showed a lower trend in the CNI + MPA + SRL + glucocorticoid group compared with the CNI + MPA + glucocorticoid group (3.6% vs. 9.1%, Figure 2). However, the difference between groups did not reach statistical significance.

### 3.4 Comparison of the blood/urine routine and biochemical indexes between groups

**FIGURE 3:**
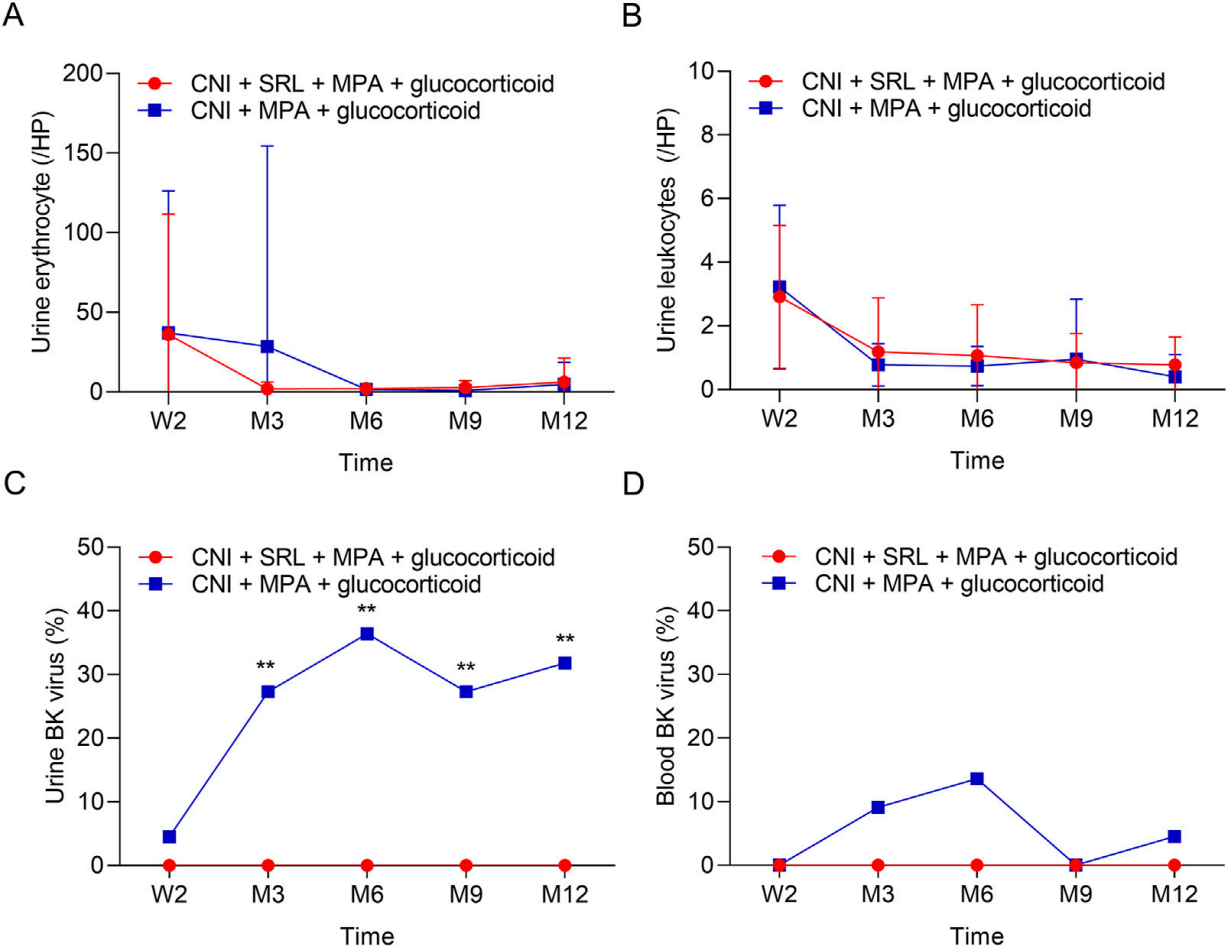
The urine indexes and BK virus rate between groups. Comparison of the urine erythrocyte **(A)**, urine leukocytes **(B)**, urine BK virus **(C)**, and blood BK virus **(D)** between the CNI + MPA + SRL + glucocorticoid and the CNI + MPA + glucocorticoid groups.

**FIGURE 4:**
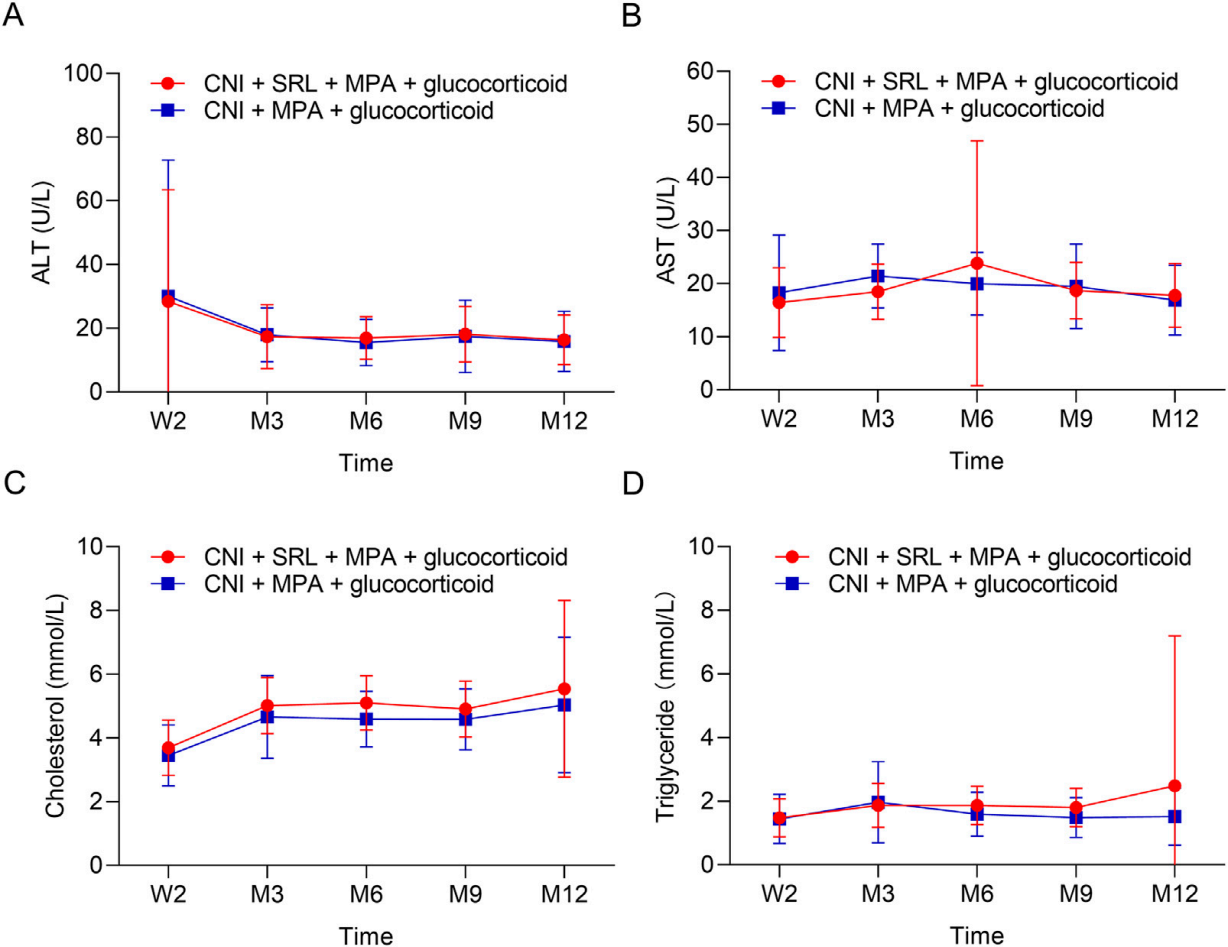
The biochemical indexes between groups. Comparison of the ALT **(A)**, AST **(B)**, cholesterol **(C)**, and triglyceride **(D)** between the CNI + MPA + SRL + glucocorticoid and the CNI + MPA + glucocorticoid groups.

**FIGURE 5:**
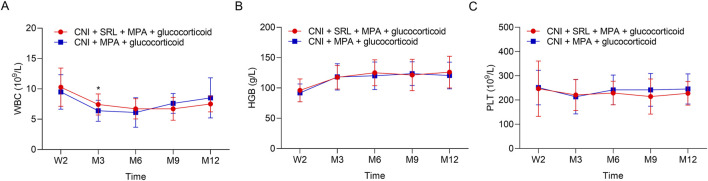
The blood routine indexes between groups. Comparison of the WBC **(A)**, HGB **(B)**, and PLT **(C)** between the CNI + MPA + SRL + glucocorticoid and the CNI + MPA + glucocorticoid groups.

The urine erythrocytes and leukocytes were similar between groups at W2, M3, M6, M9, and M12 (all *P* > 0.05, Figures 3A,B). The urine BK virus was lower in the CNI + MPA + SRL + glucocorticoid group compared with the CNI + MPA + glucocorticoid group at M3, M6, M9, and M12 (all *P* < 0.01, Figure 3C). The blood BK virus was similar between groups at W2, M3, M6, M9, and M12 (all *P* > 0.05, Figure 3D). Furthermore, the ALT, AST, cholesterol, and triglyceride were not different between groups at W2, M3, M6, M9, and M12 (all *P* > 0.05, Figures 4A–D). The WBC was higher in the CNI + MPA + SRL + glucocorticoid group compared with the CNI + MPA + glucocorticoid group at M3 (*P* < 0.05, Figure 5A), while the WBC at other timepoints, HGB at all follow-up timepoints, and PLT at all follow-up timepoints were similar between groups (all *P* > 0.05, Figures 5A–C).

### 3.5 Safety profile

During the perioperative period, the delayed graft function occurred in 2 (7.1%) patients in the CNI + MPA + SRL + glucocorticoid group and in 1 (4.5%) patient in the CNI + MPA + glucocorticoid group. Other adverse events included liver damage, infection, and poor wound healing. However, there was no difference between groups (*P* > 0.05, Table 2). During the 2 years after the operation, the most common adverse event was infection, and there was no difference between groups (35.7% vs. 22.7%). It should be noticed that the BK virus was lower in the CNI + MPA + SRL + glucocorticoid group compared with the CNI + MPA + glucocorticoid group (0.0% vs. 36.4%, *P* < 0.01, Table 2). Other adverse events, including proteinuria and diarrhea, were similar between groups (Table 2).

**TABLE 2:** Adverse events.

Items	CNI + MPA + SRL + glucocorticoid (*N =* 28)	CNI + MPA + glucocorticoid (*N =* 22)	P-value
Perioperative period			
DGF, n (%)	2 (7.1)	1 (4.5)	0.701
Liver damage, n (%)	2 (7.1)	1 (4.5)	0.701
Infection, n (%)	1 (3.6)	2 (9.1)	0.415
Poor wound healing, n (%)	0 (0.0)	3 (13.6)	0.079
2-year after operation			
Infection, n (%)	10 (35.7)	5 (22.7)	0.320
Proteinuria, n (%)	4 (14.3)	0 (0.0)	0.121
Diarrhea, n (%)	3 (10.7)	1 (4.5)	0.621
BK virus, n (%)	0 (0.0)	8 (36.4)	0.001

**Indicated *P* < 0.01 between two groups.

CNI, calcineurin Inhibitor; MPA, mycophenolic acid; SRL, sirolimus; DGF, delayed renal graft function.

## 4 Discussion

In this study, we compared two immunosuppressive regimens, CNI + MPA + SRL + glucocorticoid and CNI + MPA + glucocorticoid, to evaluate their effects on renal function, acute rejection, BK virus infection rates, and overall safety in kidney transplant recipients. Our results demonstrated that the addition of SRL to the immunosuppressive regimen offered several advantages, particularly in terms of renal function and BK virus infection rates.

In this study, the CNI + MPA + SRL + glucocorticoid group achieved higher eGFR and lower serum creatinine levels compared to the CNI + MPA + glucocorticoid group, which was consistent with previous studies (Cucchiari et al., 2020; Rodrigues et al., 2023; Tomita et al., 2023). This finding might be explained as follows: (1) As an mTOR inhibitor, the SRL would modulate immune responses and reducing the production of pro-fibrotic cytokines, therefore act as an nephroprotector (Song et al., 2021; White et al., 2025). (2) SRL could inactivate T-cells and the subsequent inflammatory response, thereby preventing tissue injury and fibrosis in the renal graft via inhibiting the mTOR pathway. Hence, the involvement of SRL to CNI contained regimen might be an optimal method to improve the kidney function in renal transplantation patients. It should be noticed that the creatinine significantly decreases on M6 and M12 in CNI + MPA + SRL + glucocorticoid groups, while eGFR significantly increases only on M12 but not on M6. The potential reason might be that: eGFR was an estimated parameter derived from serum creatinine, age, sex, etc. and minor or short-term decreased in creatinine may not immediately result in detectable changes in eGFR. Therefore, there was no difference in eGFR between groups at M6.

Besides, we also found that the incidence of acute rejection was numerically lower in the CNI + MPA + SRL + glucocorticoid group compared with the CNI + MPA + glucocorticoid group (3.6% vs. 9.1%), although there is no statistical significance. This finding could be explained as follows: (1) The small sample size limited the statistical significance leading to the statistical insignificance. (2) The duration of follow-up was too short to explicate the impact of SRL on long-term rejection outcomes. (3) Hypothesized from the previous study that SRL can reduce acute rejection by enhancing graft function and modulating immune responses (Ehx et al., 2021; Tanrısev et al., 2021; Kishimoto et al., 2023; Li et al., 2025); therefore the reduction in T-cell activation may contribute to decreased rejection episodes; however, this hypothesis needed larger studies to validate.

BK virus reactivation would vastly impact on the graft function in kidney transplant patients, which also concerns the nephrologist (Kant et al., 2022; Nakamura et al., 2024). Another finding should not be neglected that BK virus infection was lower in the CNI + MPA + SRL + glucocorticoid group compared to the CNI + MPA + glucocorticoid group. This finding would be explained as follows: SRL shows the immunosuppressive properties, and it could inhibited the viral replication by modulating T-cell responses (Liacini et al., 2010). The inclusion of SRL in the immunosuppressive regimen appeared to provide additional protection against BK virus, making it a valuable component in managing viral complications in transplant patients. This effect may be due to SRL’s ability to suppress the immune response in a way that reduces viral replication while still preventing rejection (Liacini et al., 2010). However, while the data was promising, further investigation was needed to fully understand the mechanism by which SRL affects BK virus reactivation and to confirm its role in reducing viral infections in transplant recipients. Furthermore, recent study also mentions that the medicine and food homology, which propose the possibility of adding the traditional Chinese medicine to the treatment might further improve the efficacy of SRL based regimen for anti-rejection (Law and Au, 2025). However, there is still long way to go to achieve this goal.

Finally, in terms of overall safety, our study found no significant differences between the two regimens in terms of adverse events or complications, except the BK virus infection. This suggested that the addition of SRL to the immunosuppressive regimen did not increase the risk of adverse effects when compared to the CNI + MPA + glucocorticoid regimen. This finding was consistent with previous reports on the safety of SRL in kidney transplant recipients, which have demonstrated that SRL does not lead to significant increases in the incidence of adverse events (Toniato de Rezende Freschi et al., 2024). The favorable safety profile of SRL made it an attractive option for inclusion in kidney transplant immunosuppressive protocols.

The selection of follow-up time points in this study was based on both clinical practice the pharmacological features of the study durgs. (1) The chosen of W2 was because: during the early post-transplant phase, the risk of acute rejection is high and the blood concentration would achieve a steady state; therefore, the W2 was chosen. The chosen of subsequent M3, M6, M9, and M12 was because: the graft function would be relative stable, even though there is risk of acute rejection but low, and the safety concerns as well as the blood concentration of SRL and CNI also needed periodic monitoring; therefore, the M3, M6, M9, and M12 were chosen.

Despite these promising findings, several limitations of this study need to be acknowledged. First, the relatively small sample size limited the statistical power of some of our analyses, particularly in detecting significant differences in acute rejection rates. Future studies with larger sample sizes are needed to better assess the impact of SRL on rejection outcomes. Second, the follow-up period was relatively short, which may have limited our ability to detect long-term outcomes such as chronic graft dysfunction or late-onset BK virus reactivation. Longer follow-up studies are necessary to evaluate the sustained effects of SRL on renal function and transplant outcomes. Third, this study did not assess the long-term safety profile of SRL, which remained a critical consideration when incorporating new immunosuppressive agents into clinical practice. As such, future research should explore the long-term safety and efficacy of SRL in larger cohorts of kidney transplant patients. Forth, patients were grouped according to the actual protocol they received, rather than randomly assigned, which might leading to the selection bias.

## 5 Conclusion

In conclusion, our study suggested that the CNI + MPA + SRL + glucocorticoid regimen may offer superior renal function preservation and a reduction in BK virus infection rates compared to the CNI + MPA + glucocorticoid regimen. While the addition of SRL did not significantly reduce acute rejection rates in this study, the promising trends observed warranted further investigation. Additionally, the overall safety profile of SRL was favorable, supporting its potential use in kidney transplant immunosuppressive regimens. However, larger, longer-term studies are needed to confirm these findings and better understand the role of SRL in improving long-term transplant outcomes.

## Data Availability

The original contributions presented in the study are included in the article/supplementary material, further inquiries can be directed to the corresponding author.
